# FGFR1 and NTRK3 actionable alterations in “Wild-Type” gastrointestinal stromal tumors

**DOI:** 10.1186/s12967-016-1075-6

**Published:** 2016-12-14

**Authors:** Eileen Shi, Juliann Chmielecki, Chih-Min Tang, Kai Wang, Michael C. Heinrich, Guhyun Kang, Christopher L. Corless, David Hong, Katherine E. Fero, James D. Murphy, Paul T. Fanta, Siraj M. Ali, Martina De Siena, Adam M. Burgoyne, Sujana Movva, Lisa Madlensky, Gregory M. Heestand, Jonathan C. Trent, Razelle Kurzrock, Deborah Morosini, Jeffrey S. Ross, Olivier Harismendy, Jason K. Sicklick

**Affiliations:** 1 School of Medicine, University of California San Diego, La Jolla, CA USA; 2 Foundation Medicine, Inc., Cambridge, MA USA; 3 Oncogenomics Laboratory, Division of Biomedical Informatics, Moores UCSD Cancer Center, UC San Diego Health Sciences, University of California San Diego, 3855 Health Sciences Drive, Room 4335, Mail Code 0820, La Jolla, CA 92093-0820 USA; 4 Portland VA Health Care System, Portland, OR USA; 5 Knight Cancer Institute, Oregon Health Sciences University, Portland, OR USA; 6 Department of Pathology, Sanggye Paik Hospital, Inje University, Seoul, Korea; 7 Division of Cancer Medicine, Department of Investigational Cancer Therapeutics, The University of Texas MD Anderson Cancer Center, Houston, TX USA; 8 UCSD Department of Radiation Medicine and Applied Sciences, Moores UCSD Cancer Center, University of California San Diego, La Jolla, CA USA; 9 Division of Medical Oncology, Department of Medicine, Moores UCSD Cancer Center, University of California San Diego, La Jolla, CA USA; 10 Department of Medical Oncology, Fox Chase Cancer Center, Philadelphia, PA USA; 11 UCSD Department of Family and Preventive Medicine, Moores UCSD Cancer Center, University of California San Diego, La Jolla, CA USA; 12 Sarcoma Medical Oncology Program, University of Miami Sylvester Cancer Center, Miami, FL USA; 13 Division of Surgical Oncology, Department of Surgery, Moores UCSD Cancer Center, UC San Diego Health Sciences, University of California San Diego, 3855 Health Sciences Drive, Room 2313, Mail Code 0987, La Jolla, CA 92093-0987 USA

**Keywords:** Gene sequencing, Mutation, GIST, FGFR1, ETV6–NTRK3

## Abstract

**Background:**

About 10–15% of adult, and most pediatric, gastrointestinal stromal tumors (GIST) lack mutations in KIT, PDGFRA, SDHx, or RAS pathway components (KRAS, BRAF, NF1). The identification of additional mutated genes in this rare subset of tumors can have important clinical benefit to identify altered biological pathways and select targeted therapies.

**Methods:**

We performed comprehensive genomic profiling (CGP) for coding regions in more than 300 cancer-related genes of 186 GISTs to assess for their somatic alterations.

**Results:**

We identified 24 GIST lacking alterations in the canonical KIT/PDGFRA/RAS pathways, including 12 without SDHx alterations. These 24 patients were mostly adults (96%). The tumors had a 46% rate of nodal metastases. These 24 GIST were more commonly mutated at 7 genes: ARID1B, ATR, FGFR1, LTK, SUFU, PARK2 and ZNF217. Two tumors harbored FGFR1 gene fusions (FGFR1–HOOK3, FGFR1–TACC1) and one harbored an ETV6–NTRK3 fusion that responded to TRK inhibition. In an independent sample set, we identified 5 GIST cases lacking alterations in the KIT/PDGFRA/SDHx/RAS pathways, including two additional cases with FGFR1–TACC1 and ETV6–NTRK3 fusions.

**Conclusions:**

Using patient demographics, tumor characteristics, and CGP, we show that GIST lacking alterations in canonical genes occur in younger patients, frequently metastasize to lymph nodes, and most contain deleterious genomic alterations, including gene fusions involving FGFR1 and NTRK3. If confirmed in larger series, routine testing for these translocations may be indicated for this subset of GIST. Moreover, these findings can be used to guide personalized treatments for patients with GIST.

*Trial registration* NCT 02576431. Registered October 12, 2015

**Electronic supplementary material:**

The online version of this article (doi:10.1186/s12967-016-1075-6) contains supplementary material, which is available to authorized users.

## Background

Gastrointestinal stromal tumors (GISTs) are the most common sarcoma affecting approximately 3500 new patients per year in the United States [1]. Approximately 70–80% of sporadic GISTs are caused by gain-of-function mutations in KIT (c-KIT, CD117); another 5–10% are caused by activating genomic alterations in platelet-derived growth factor receptor alpha (PDGFRA) [2–4]. Even less common are tumors driven by RAS pathway gene mutations (e.g., BRAF, KRAS, NF1; 1–3% combined) or mutations/deficiencies in the four succinate dehydrogenase (SDH subunits (A, B, C, or D; 3% combined) [2, 3, 5–8].

Given the initial understanding that the majority of GISTs were driven by KIT, the application of targeted drugs such as imatinib mesylate (Novartis, Basel, Switzerland) allowed GIST to serve as the paradigm for cancer genotyping and the development of “matched” therapies for solid malignancies. Imatinib and other small molecule tyrosine kinase inhibitors have demonstrated clear anti-GIST activity by targeting oncogenic KIT and PDGFRA mutations, and as a result, these drugs are now firmly established in treatment of GIST patients [9].

However, approximately 5–15% of adult GIST patients, and most pediatric patients, who tend to be imatinib-resistant, were initially designated as “wild-type (WT)” until other oncogenic mutations were identified. Some have termed this subset of patients “quadruple-WT (qWT)”, or “quadruple-negative GISTs” because they lack oncogenic mutations in any of the aforementioned genes [10]. Since the designation of qWT GIST, only one study has attempted to define and compare its molecular profile to other GISTs, reporting the overexpression of polycomb target genes (e.g., CDK6, ERG and NTRK2) as potential drivers and identifying potential diagnostic markers (e.g., CALCRL and COL22A1) [11]. This study provided insight into alternative pathways that may be involved in GIST oncogenesis; however, this report was limited by analyzing only two so-called qWT tumors. Beyond this study, there is a lack of reports addressing the clinical demographics and molecular characteristics of qWT GISTs [10]. Thus, the patient demographics, pathological data (i.e., tumor location), and genomic profile of qWT GISTs are largely unknown. These deficiencies in our knowledge leave affected patients at a disadvantage by not identifying the genetic abnormalities that could be used to diagnose GIST and delay the development of novel and potentially beneficial precision therapies for qWT GIST.

Given the increasing application of comprehensive genetic profiling (CGP) towards personalized medicine treatments in oncology, we hypothesized that analyzing demographic data, pathological data, and genomic profiles of quadruple-WT GIST would begin to define the pathobiology of this largely undefined GIST subtype, as well as reveal clinically relevant, potentially targetable, genomic alterations (GAs).

## Methods

### Primary study population

The Foundation Medicine, Inc. (Cambridge, MA, USA) database consists of patients from across the world treated in both academic and private practice settings. De-identified tumor samples were selected from the database of patients who underwent Foundation One™ analyses in the course of clinical care from October 2012 to May 2015, where 186 tumors were categorized as GISTs according to pathologists at the diagnosing institutions. Demographic and clinicopathologic data were compared to population-based data (N = 6112) from the National Cancer Institute’s Surveillance, Epidemiology, and End Results (SEER) database with histologically-confirmed GIST between 2001 and 2011 [1].

### Study design

Patient demographic and tumor clinicopathologic data included age and sex, primary GIST site, tumor biopsy site, and TNM disease stage. We retrospectively analyzed this prospectively collected data under a University of California, San Diego Institutional Review Board approved protocol (#141555X). An experienced pathologist (JSR) centrally re-reviewed hematoxylin and eosin-stained sections of 29 tumors lacking driver mutations in KIT, PDGFRA, or RAS pathway components (BRAF, NF1, KRAS, HRAS, NRAS). Additionally, 12 tumors lacked driver mutations in SDHx. Five tumors were excluded as more consistent with other sarcoma subtypes.

### Comprehensive genomic profiling

GIST tumor specimens were taken from either primary or secondary tumor biopsies and submitted to a Clinical Laboratory Improvement Act certified, College of American Pathologists and New York State accredited laboratory, Foundation Medicine, Inc. (FMI), by healthcare providers from various medical institutions. Comprehensive genomic profiling (CGP) was performed on hybridization-captured, adaptor ligation-based libraries using DNA extracted from four formalin-fixed paraffin embedded (FFPE) sections cut at 10 µm from the tumors. All samples sent for DNA extraction contained a minimum of 20% tumor nuclei. The FoundationOne™ assay is a next-generation sequencing-based genomic assay that utilizes the Illumina HiSeq 2500 instrument (Illumina Inc., San Diego, CA, USA) to sequence coding regions of more than 182 cancer related genes (including SDHx for 109 patients). The number of genes in the FoundationOne™ panel has evolved over time as new data on cancer-related genes has been published. But, all versions of the assay simultaneously analyze the extracted DNA for base substitutions, short insertions and deletions, amplifications and homozygous deletions and gene rearrangements as previously described [12].

### Genomic analysis

The genomic alterations were identified using Foundation Medicine workflow [12]. To maximize sensitivity in heterogeneous GIST specimens, the test was validated to detect base substitutions, as well as short insertions and deletions at ≥10% mutant allele frequency with ≥99% sensitivity, The genomic alterations were further categorized [i.e., known somatic, likely somatic, or variant of unknown significance (VUS)] according to Foundation Medicine classification [12]. Furthermore, point mutations and small indels were annotated for their functional effect on the protein (missense, nonsense, frameshift, etc.).

In order to expand our understanding of the potential deleterious effect of missense VUSs, we mapped them to the dbNSFP database, which pre-computed multiple scores for all possible base substitutions in coding regions [13, 14]. VUSs that were predicted deleterious by at least 2 out of 4 prediction tools (SIFT, PolyPhen, MutationTaster, and/or MutationAssessor) were included in further analyses (Additional file 1: Table S1). Any missense VUS that did not meet this criterion was excluded and not reported.

As the FMI sequencing process does not include sequencing of a matched normal tissue for germline analysis, it is possible for predicted-deleterious inherited variants in cancer related genes to be detected and included in the reports with somatic variants. Thus, we sought to exclude common non-SDH [A-D] and non-NF1 germline alterations in order to focus on putative somatic alterations. The Exome Aggregation Consortium (ExAC) Browser (https://exac.broadinstitute.org) provides a reliable registry of all known human germline variation down to a minor allele frequency of 10^−5^ [15]. We were able to match 495/1605 (30.8%) of the short variants to the same residue, as well as with the same amino-acid substitution in the ExAC database (version 0.3) using the protein annotation. Mutations present in the ExAC database at a minor allele frequency greater than 1% (i.e., NOTCH2, FANCD2, MAP3K1, MSH3, and ZNF217), indicating potential germline variants, were also excluded from analysis (Additional file 1: Table S2).

### Secondary study population

To confirm our results, we identified 5 additional cases of qWT GIST after clinical testing at the Knight Diagnostic Laboratory (OHSU Knight Cancer Institute, Portland, OR, USA). All of these cases had been previously screened for mutations in genes known or thought to be involved in GIST biology using a custom panel including hotspots or entire coding regions of AKT1, AKT2, AKT3, ATM, BRAF, CDKN2A, HRAS, KIT, KRAS, MAP2K1, NF1, NRAS, PDGFRA, PIK3CA, PTEN, PTPN11, SDHA, SDHAF1, SDHAF2, SDHB, SDHC, SDHD and TP53. Library preparation, sequencing, and variant calling were carried out using reagents, instruments and software from Thermo Fisher Scientific, as previously described [16]. Libraries were amplified by emulsion PCR on ion sphere particles (ISPs) using an Ion Xpress Template Kit. The templated ISPs were recovered from the emulsion, and enriched with MyOne streptavidin C1 Dynabeads. Eight barcoded samples were multiplexed on an Ion 318 chip and sequenced on a PGM sequencer (Ion PGM200 sequencing kit). Torrent Suite software version 4.0 was used to parse barcoded reads, to align reads to the reference genome, and to generate run metrics, including chip loading efficiency and total read counts and quality. Variants were identified with Variant Caller software version 4.0, and target coverage was evaluated with Coverage Analysis software version 4.0 [16]. Amplicon sequencing libraries were prepared from 20 ng of RNA purified from residual DNA from previous clinical testing using the Ion Total RNA-Seq kit (Thermo Fisher Scientific) according to the manufacturer’s instructions. These libraries were subjected to analysis using a panel that included primer pairs for 169 known gene fusions that involve target genes and 94 fusion partners (Additional file 1: Table S3) as previously described [17].

## Results

### Demographic and clinicopathologic tumor data

The vast majority of tumors (87.1%, 162/186) contained mutations in known or suspected drivers of GIST (Table 1). In contrast, we identified 24 (13%) patients with WT tumors. This included 12 tumors identified as KIT^WT^/PDGFRA^WT^/BRAF^WT^/SDH^WT^/NF1^WT^/KRAS^WT^/HRAS^WT^/NRAS^WT^ or qWT GIST and 12 tumors identified as KIT^WT^/PDGFRA^WT^/BRAF^WT^/NF1^WT^/KRAS^WT^/HRAS^WT^/NRAS^WT^ GIST with unknown SDH status.

**Table 1:** Identification of quadruple wild-type GIST (qWT) Subset in 186 GIST

Category	Number of patients^a^	Percent of patients (%)
KIT mutated	129	69
PDGFRA mutated	22	12
NF1 mutated	18	10
SDH [ABCD] mutated	14	8
BRAF mutated	7	4
[KNH] RAS mutated	4	2
qWT	12	6
SDH unknown	12	6

^a^Genomic alterations are not mutually exclusive and are potentially germline (e.g., NF1 and SDH [ABCD]). Of the 162 non-WT GISTs, 32 tumors had one or more genomic alterations in KIT, PDGFRA, NF1, SDH [A-D], BRAF, and/or [KNH] RAS

Demographic and clinicopathologic data for these 24 patients are provided in Additional file 1: Table S4. In the WT GIST subset, the average age at diagnosis was 44.4 ± 15.7 (median 45 years old) with equal gender distribution. The tumor samples were obtained from primary tumors (N = 15, 62.5%), metastatic sites (N = 6, 25%), or unknown abdominal sites (N = 3, 12.5%). The stomach was the most common primary site (N = 13, 54.2%), similar to SEER data (P = 0.23). All patients had tumors greater than 2 cm. The WT cohort mainly consisted of tumors that are >5 and ≤10 cm (66.7%). An additional 8.3% were smaller and 20.8% were larger. Data on mitotic index was not available. In addition, the WT cohort consisted of 45.8% with lymph node metastases (N1) and 70.8% with distant metastases (M1). Thus, WT GISTs occurred throughout the GI tract, tended to be large in size (>5 cm), and frequently had nodal and/or distant metastases.

### Genomic alterations in GIST

In the 186 GISTs studied, CGP revealed a total of 1385 unique alterations (1276 short variants, 83 copy number alterations and 26 rearrangements) affecting all 186 GIST patients (Additional file 1: Figure S1; Table S5). The cohort assembled reflects, at the genomic level, the current knowledge of sporadic GIST in the U.S. Genomic alterations in the canonical genes affected 162 GIST (referred to as non-WT) were not mutually exclusive as has been previously reported [18]. Indeed, 32 non-WT tumors had two or more genomic alterations in KIT, PDGFRA, NF1, SDH [A-D], BRAF, and/or [KNH]RAS (Additional file 2: Table S6). Overall, 129/186 (69%) of GISTs had a KIT alteration. Similarly, PDGFRA was the second most commonly mutated canonical gene with 22 of 186 (12%) cases. Of the subset of 97 non-WT GIST tested for SDH, 14 (14.4%) had a mutation detected, 6 of them in absence of any mutations in KIT, PDGFRA or RAS pathways.

**Fig. 1:**
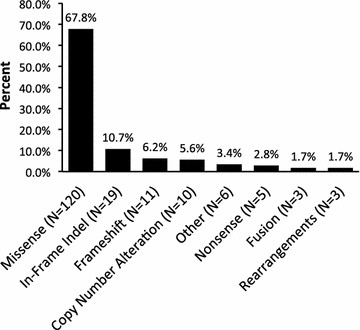
Types of Genomic Alterations Detected on Broad Genetic Profiling of Wild-Type GIST. Bar graph demonstrates the types of genomic alterations identified in WT GIST as determined by CGP). Percentages and total numbers (N) of mutations are indicated

In the 24 tumors designated as WT (i.e., KIT^WT^/PDGFRA^WT^/BRAF^WT^/SDH^WT/UNKNOWN^/NF1^WT^/HRAS^WT^/NRAS^WT^KRAS^WT^), CGP analyses revealed an average of 7.6 ± 3.2 genomic alterations (range: 3–17). Over the entire WT cohort, 120 genes are altered; the alterations include 120 missense mutations, 19 in-frame indels, 11 frameshifts, 10 copy number alterations (6 amplifications and 4 losses, 6.4%), 5 nonsense mutations, 3 gene fusions, 3 rearrangements, and 6 unclassified mutations (Fig. 1). The affected genes and their respective alterations are listed in Additional file 3: Table S7.

**Fig. 2:**
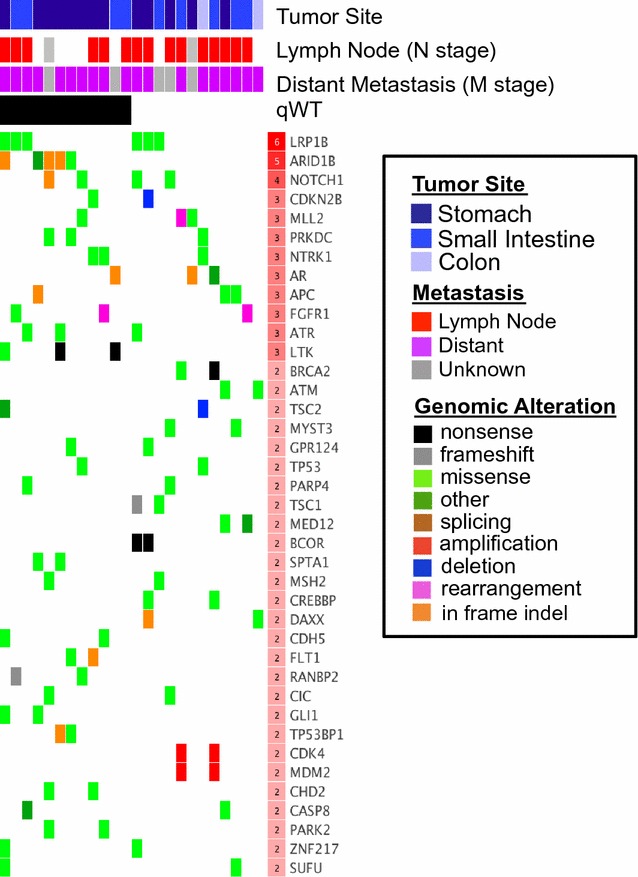
Deleterious genomic alterations, genes and tumor sites in Wild-Type GIST. Matrix demonstrating genes recurrently mutated in WT GIST patients, with each column representing an individual patient. VUS missense mutations are displayed only if they are predicted to affect gene function by 2 or more algorithms (see “Methods” section). Genes were prioritized on the basis of predicted damaging effect. The table header indicates GIST tissue of origin (*blue*), positive node status (*red*), positive metastic status (*purple*), qWT status (i.e. tested for SDHx—*black*) missing data (*grey*). The number of mutated patients for each gene (*red scale* in row header) is indicated

There are 39 genes recurrently mutated in the WT samples (Fig. 2). The genes most commonly altered in WT tumors were LRP1B (N = 6), ARID1B (N = 5), and NOTCH1 (N = 4). Importantly, these genes are also recurrently mutated in qWT alone. LRP1B, the most frequently mutated gene in qWT GIST, is an LDL receptor-related protein involved in hepatic metabolism, tissue remodeling, and cellular migration. Its cytoplasmic domain is demonstrated to interact with components of the PKC and RAS-MAPK pathways [19]. Evidence is mounting that LRP1B may function as a broad-spectrum tumor suppressor, with dysfunction potentially linked to increased tumor invasion and chemotherapeutic resistance [20–23]. LRP1B mutations may also be associated with upregulation of inflammatory responses, consistent with a growing body of evidence that chronic inflammation predisposes to cancer formation [24].

**Fig. 3:**
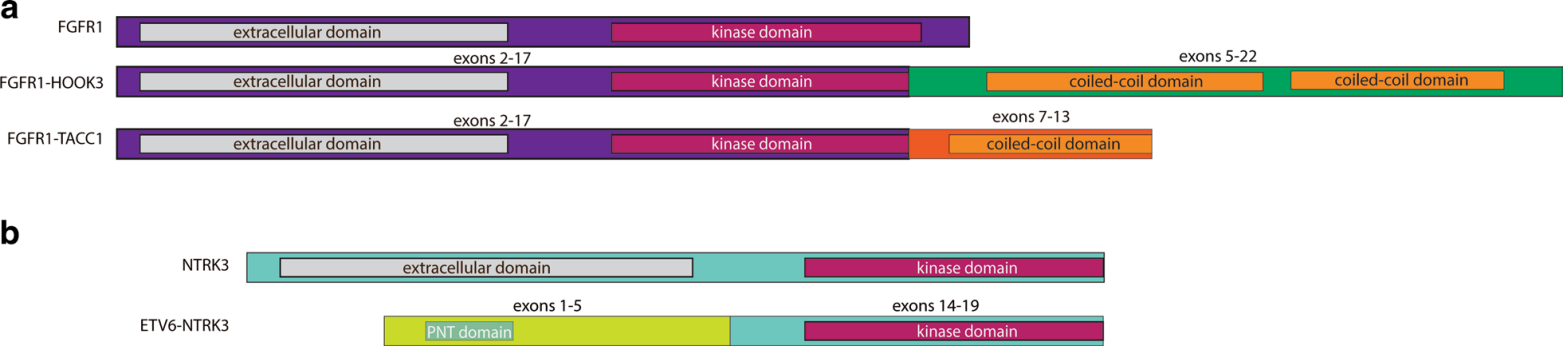
Kinase fusions identified in WT GIST samples. Three separate fusions involving the N-terminus of FGFR1 (**a**) and the C-terminus of NTRK3 (**b**) were identified. The FGFR1 fusions (**a**) were similar in structure to reported fusions and contained exons 2-17 fused with exons 5-22 of HOOK3 or exons 7-13 of TACC1. Intact coiled-coil motifs were present in both fusion partners and likely facilitate dimerization. Note that exon 1 of FGFR1 is non-coding and therefore excluded from the protein diagrams. The NTRK3 fusion (**b**) contained exons 1-5 of ETV6 and exons 14-19, which included the complete kinase domain. Although the portion of ETV6 present in the fusion lacked the DNA-binding domain, a Pointed (PNT) was conserved. This region is composed of 5-helix bundle involved in protein–protein interactions and may facilitate dimerization of this fusion. All diagrams are drawn to scale

In order to identify potential drivers specific to WT GIST, we determined whether the recurrently mutated genes were more likely to be mutated in WT than in non-WT GIST. We identified 7 genes significantly more mutated in WT patients (P < 0.05): LTK, SUFU, ZNF217, ARID1B, PARK2, ATR, and FGFR1 (Table 2). The FGFR1 gene is altered with 1 missense mutation and 2 gene fusions including FGFR1–HOOK3 (predicted activating in-frame fusion of FGFR1 intron 17 and HOOK3 intron 4) and FGFR1–TACC1 [25] (predicted activating in-frame fusion of FGFR1 intron 17 and TACC1 intron 6) (Fig. 3a). All three FGFR1 alterations identified in WT GIST are known to be deleterious (K656E, FGFR1-HOOK3 and FGFR1-TACC1 fusions) in contrast to 1/5 FGFR1 alterations in the non WT GIST (amplification—Additional file 1: Table S8), thus suggesting that the FGFR1 alterations are more likely drivers in the WT GIST. PARK2 is a ubiquitin ligase regulating cyclin stability during G1 to S cell-cycle progression. ATR is a subunit of a double-stranded break DNA repair complex. ARID1B is a chromatin remodeling factor that may play a role in suppressing the oncogenic Wnt/β-catenin pathway [26–30]. SUFU is a negative regulator of Hedgehog signaling pathways, which have been implicated in directing mesenchymal growth within the GI tract and, when overexpressed, in GI tumor carcinogenesis [31, 32]. Both ARID1B and SUFU may be actionable via FDA-approved histone deacetylase (HDAC) inhibitors and Hedgehog pathway inhibitors, respectively. ZNF217 is a transcription factor associated with poor prognosis in various carcinomas, especially increased metastatic potential in colorectal cancer [33, 34]. Other recurrently mutated genes of interest include APC, BCOR, CDK4, MDM2, and TP53 (N = 2 each). Of note, both tumors with alterations in BCOR (BCL6 Corepressor) had nonsense mutations and were gastric GISTs. BCOR is a gene which is implicated in B cell activation and may be an example of a link between inflammation and GIST [35, 36]. Studies of investigational drug RI-BPI, which inhibits BCL6 by abrogating its interaction with BCOR, show efficacy against diffuse large B-cell lymphomas (DLBCL) with BCL6, representing yet another potential targeted therapy for specific qWT patients [37, 38]. Moreover, by combining EZH2 histone methyltransferase inhibitors with RI-BPI, there have been preliminary reports of synergism.

**Table 2:** Genes significantly more affected in wild-type GIST

Gene	Alterations in non-WT (%)	Alterations in WT (%)	P value
LTK	2 (1.2 %)	3 (12.5%)	0.01602
SUFU	0 (0 %)	2 (8.3%)	0.01604
ZNF217	0 (0 %)	2 (8.3%)	0.01604
ARID1B	11 (6.8 %)	5 (20.8%)	0.03826
PARK2	1 (0.6 %)	2 (8.3%)	0.04429
ATR	4 (2.5 %)	3 (12.5%)	0.04689
FGFR1	4 (2.5 %)	3 (12.5%)	0.04689

For the first time, we discovered two novel FGFR1 kinase fusions in GIST, as well as identified a third gene fusion that was reported during the preparation of this manuscript [39]. These included the aforementioned FGFR1–HOOK3 fusion in a 38-year old female with a T3N1M1 small bowel GIST and the FGFR1–TACC1 fusion in a 54-year old male with a T3N1M1 gastric GIST, as well as an ETV6–NTRK3 fusion in a 55-year old male with a T3N0M1 small bowel GIST (Fig. 3). A total of three patients possessed alterations in FGFR1, including a 60-year old male with a T3NxM1 small intestine GIST with a known FGFR1 missense mutation. Their respective FGFR1 alterations were their only known/likely alterations identified, suggesting a high likelihood of being driver alterations. Of note, an additional 72-year old male with a T4N1Mx small intestine GIST possessed a FGF6 amplification, among multiple other GAs, potentially having the same functional impact on tumor growth since FGF6 is a reported ligand of FGFR1 [40, 41].

To increase the possibility of detecting gene fusions resulting from intronic breakpoints that would be missed with our DNA-based sequencing panel, we investigated 5 additional cases of qWT GIST collected from an alternate clinical testing laboratory (“Methods” section). These included 3 tumors known to express SDHB and 2 tumors that were without SDH gene mutations but whose SDHB protein expression status (as assessed by immunohistochemistry) was unknown. Demographic and clinicopathologic data for these 5 patients are provided in Additional file 1: Table S9. These cases were additionally analyzed using an RNA-based sequencing panel that included primer pairs for 169 known gene fusions that involve target genes and 94 fusion partners (Additional file 1: Table S3). We identified one additional case with an FGFR1-TACC1 fusion and one additional case with an ETV6-NTRK3 gene fusion.

### Clinically relevant genomic alterations

**Fig. 4:**
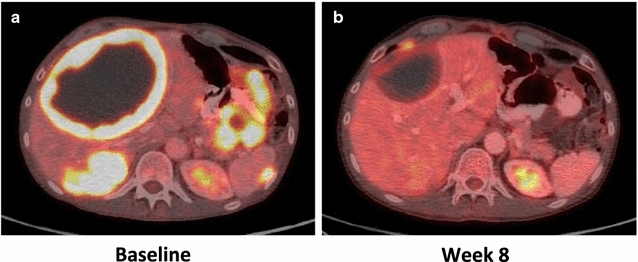
Radiological response of a GIST possessing an ETV6–NTRK3 fusion following treatment with LOXO-101, a selective TRK inhibitor. A 55-year old male with a T3N0M1 small intestine GIST had progression of disease on five lines of tyrosine kinase inhibitors targeting KIT prior to identification of an ETV6–NTRK3 fusion in the tumor. He was enrolled on a Phase I clinical trial of oral LOXO-101 (Loxo Oncology, Stamford, CT), a selective TRK inhibitor. As compared to baseline PET/CT images (**a**), the tumors had decreased size and FDG-uptake at week 8 (**b**). At 4 months, the patient had ongoing partial response (44%) according to RECIST 1.1 criteria

At present, many of the identified genomic alterations are not clinically actionable or known to be activating genetic events. However, alterations in a subset of genes may be targetable by off-label use of several FDA-approved or investigational targeted therapies. The patient we identified as having a tumor harboring an ETV6–NTRK3 fusion was a 55-year old male with T3N0M1 small intestine GIST who was treated with oral LOXO-101 (Loxo Oncology, Stamford, CT, USA), the only selective TRK inhibitor in clinical development, in a Phase I trial (NCT 02122913) [42]. The patient was originally diagnosed in May 2003 and previously progressed on five lines of therapy, including imatinib (FDA-approved first-line agent for GIST), sunitinib (FDA-approved second-line agent for GIST, Pfizer), sorafenib (Bayer), nilotinib (Novartis), and regorafenib (FDA-approved third-line agent for GIST; Bayer). At the time of study entry, the patient had significant pain. Upon receiving LOXO-101, the patient noted immediate improvement in his symptoms. Tumor response to LOXO-101 was seen at the end of week 8 by PET/CT (Fig. 4). Following 4 months of therapy, the patient had an ongoing partial response (44%) according to RECIST 1.1 criteria. Of note, this patient’s tumor also possessed likely deleterious alterations in PAX5 and SETD2. LOXO-101 is now being studied in a worldwide Phase II basket trial enrolling patients with solid tumors that harbor NTRK1/2/3 fusions (NCT 02576431). It is important to note that this symptomatic and radiologic response is in stark contrast to the 54-year old male with colonic GIST in the secondary study population who also possessed an ETV6–NTRK3 fusion. The latter patient was heavily pre-treated with progressive disease on imatinib (3 months), sunitinib (2 months), sorafenib (2 months), and linsitinib (5 months, OSI Pharmaceuticals). The identification of the ETV6–NTRK3 fusion earlier in the disease course may have altered the treatment choices and outcome.

## Discussion

We report the identification of clinically relevant and previously unreported genomic alterations in gastrointestinal stromal tumors (GISTs) lacking genomic alterations in KIT, PDGFRA, SDH, and the RAS pathway. We profiled patient demographics and tumor characteristics of our cohort and performed CGP of tumors on this subset of GIST patients, including reporting of clinical responses to molecularly matched therapies. Our findings suggest new and potentially targetable alterations in genes such as NTRK3 and FGFR1 in a subset of GIST patients.

The current study focuses on a patient population that we are just beginning to understand more thoroughly. Even without performing whole exome or whole genome sequencing, our study findings expand upon the few reports of the molecular characteristics of WT GISTs [7, 10, 11, 39, 43]. We compare the incidence of genomic alterations in WT and non-WT GIST, yielding seven genes that appear to be more commonly altered in WT GIST. This includes LTK (lymphocyte receptor tyrosine kinase) and FGFR1, two non-KIT/PDGFRA receptor tyrosine kinases that mainly signal through the RAS-MAPK pathway. In addition, LTK also signals through the PI3K-AKT-mTOR pathway in order to maintain survival signals in tumor cells. These genomic alterations detected in WT GIST affect key pathways such as the PI3K-AKT-mTOR and RAS-MAPK pathways, which overlap with downstream signaling of several known drivers, including KIT and PDGFRA. In addition to cell-cycle regulation, other genomic alterations were seen to affect histone acetyltransferases, transcriptional regulators, and the NFkB pathway, as well as embryonic development and cancer stem cell pathways (e.g., Wnt/β-catenin pathway and Notch pathway). Taken together, the genomic profiles highlight novel genes in WT GIST with similar or intersecting functions as known drivers of non-WT GIST development. Moreover, several of these genomic alterations have potential therapeutic importance. Of note, five mutations in ARID1B, which is part of the SWI/SF chromatin remodeling complex, may be targetable with FDA-approved HDAC inhibitors, including vorinostat and panobinostat. Furthermore, this study uncovered mutations in SUFU, a negative regulator of the Hedgehog signaling pathway. Because SUFU is downstream of the SMO oncogene, FDA-approved agents, which target SMO (e.g., vismodegib and sonidegib) are unlikely to be effective in tumors with downstream SUFU alterations. However, we recently reported that the GLI-family of transcription factors, which are downstream of SUFU, may be targetable in GIST with the FDA-approved agent, arsenic trioxide [44]. Thus, our new findings have potential therapeutic implications for several subsets of WT GIST patients they may be treated with currently available drugs or those under development.

We detected an FGF6 mutation and four potentially actionable FGFR1 alterations, including one FGFR1 missense mutation, as well as three FGFR1 fusions including a novel FGFR1–HOOK3 fusion and two cases with an FGFR1–TACC1 fusion previously reported in glioblastoma multiforme. These events are predicted to result in constitutive activity of FGFR1 and the downstream tyrosine kinase cascades that promote WT oncogenesis perhaps in the presence or absence of growth factors (e.g., FGF6) or FGFR1 overexpression [45, 46]. Thus, a subset of GIST, that is similar in size to RAS mutant GISTs in the cohort, may be sensitive to targeted FGFR tyrosine kinase inhibitors, such as lenvatinib (Esai), ponatinib (Ariad), pazopanib (Novartis) or other similar investigational drugs.

The only prior study defining and comparing genomic profiles of 2 qWT GIST to non-qWT GISTs did not identify any genomic alterations in the two tumors but reported mRNA overexpression of polycomb target genes including CDK6, ERG and NTRK2 [11]. Interestingly, we identified genomic alterations in a related gene, NTRK3 (fusion with ETV6). ETV6–NTRK3 fusions have been reported in infantile fibrosarcoma, secretory breast carcinoma, salivary gland tumors (acinic cell carcinomas, cystadenocarcinomas, and adenocarcinomas), mixed epithelial and stromal tumor of the kidney, leukemias, and thyroid cancer. During the preparation of this manuscript, another group reported an ETV6–NTRK3 fusion in GIST [39]. These findings have clinical relevance as recent data (including that shown here) suggest that NTRK fusions are sensitive to LOXO-101. Loxo Oncology has reported two other NTRK fusion patients with clinical responses on its Phase I trial (i.e., sarcoma with LMNA–NTRK1 and mammary analogue secretory carcinoma of the salivary gland with ETV6–NTRK3) [42]. Another report suggests that crizotinib (Pfizer, New York, NY, USA) may be a treatment option for patients with NTRK fusions [47, 48]. The potential clinical applicability underscores the significant contributions that genomic profiling can add towards clinical decision making and precision therapies.

The strength of the current study includes the large sample cohort and a careful filtering of the mutation identified. The genomic data was evaluated through two different processes, namely bioinformatic analyses associated with the Foundation One™ assays and comparison to dbNSFP, in order to identify all potentially relevant findings. Foundation Medicine assays include multiple gene fusions, which have not been throughly investigated by previous studies. This allowed us to identify deleterious, actionable alteration in FGFR1 and NTRK3 in three patients. Foundation Medicine assays however cannot distinguish rare germline variants from somatic mutations, leaving some ambiguity in the findings specifically in regards to rare germline SDH and NF1 mutations. Finally the CGP approach only screened against genes with known associations with any solid or hematologic cancer, potentially failing to capture truly novel genes that have not yet been linked to cancer development.

Given that 12 patients did not have SDHx mutation testing, we are unable to definitively determine the proportion of these patients that harbor SDHx mutations or are truly WT. Finally, due to the nature of this study, we are unable to match germline and somatic sequencing data in patients, repeat testing with the lattest gene panel that includes SDHx subunits, perform SDHB immunostaining to assess SDHB-competence/-deficiency, or assess for SDHC-epimutant tumors with hypermethylation of the SDHC promoter, which can lead to silencing of expression [7]. Despite the limitations of this study, we begin to suggest that 17 qWT GIST are biologically distinct from than their non-WT counterparts, providing novel insight into the clinico-pathological features of WT GIST.

## Conclusions

In summary, this study builds upon previous work in the GIST field and provides the new insights into the genomic landscape of quadruple-WT GIST. While these tumors historically are considered “wild-type” mainly due to a lack of KIT or PDGFRA mutations, this study showed that the majority of these tumors harbor deleterious genomic alterations in genes participating in crucial cellular activities, such as cell cycle progression, DNA repair, and regulation of gene expression. Alternatively, it is possible that the activity of the canonical genes (i.e., KIT, PDGFRA, KRAS, NF1, BRAF, SDHx, KRAS) may also be altered in WT tumors via epigenetic changes as seen in SDH-deficient tumors. Furthermore, this study identified several actionable mutations, including two ETV6–NTRK3 fusions, and FGF6 or FGFR1 alterations, including three FGFR1 fusions and one known intragenic activating FGFR1 mutation, that may significantly impact tumor responses by assisting in the choice of targeted therapies. Such findings have the potential to change present therapeutic options in GIST, give insight into disease biology, and redefine one of the earliest paradigms in tumor genomics and precision medicine. By providing novel insight into potential genetic drivers for GIST, future studies may further build on this genetic profile of so-called qWT GIST, which are not truly WT, as well as link genomic drivers to therapeutic regimens. In turn, this may lead to individualized treatments that can significantly improve patient outcomes.

## Additional files

**Additional file 1.** Additional tables and figure.

**Additional file 2: Table S6.** List of Mutations Identified in non-WT Patients. Complete tabulation of genomic alterations found in non-WT patients, with VUSs filtered for deleterious effects according to Additional files 1 and 2.

**Additional file 3: Table S7.** List of Mutations Identified in WT Patients. Complete tabulation of genomic alterations found in study cohort, with VUSs filtered for deleterious effects according to Additional files 1 and 2.

## Abbreviations

GIST: gastrointestinal stromal tumor
WT: wild-type
qWT: quadruple wild-type
tWT: triple wild-type
CGP: comprehensive genomic profiling
GA: genomic alteration
VUS: variant of unknown significance
FFPE: formalin-fixed paraffin embedded
ISP: ion sphere particle
ExAC: Exome Aggregation Consortium
FMI: Foundation Medicine, Inc.
SEER: surveillance, epidemiology, and end results

## References

[1] Ma GL, Murphy JD, Martinez ME, Sicklick JK (2015). Epidemiology of gastrointestinal stromal tumors in the era of histology codes: results of a population-based study. Cancer Epidemiol Biomarkers Prev.

[2] Corless CL, Barnett CM, Heinrich MC (2011). Gastrointestinal stromal tumours: origin and molecular oncology. Nat Rev Cancer.

[3] Rubin BP, Heinrich MC, Corless CL (2007). Gastrointestinal stromal tumour. Lancet.

[4] Fanta PT, Sicklick JK, Betz BL, Peterson MR (2015). In Vivo Imatinib Sensitivity in a Patient With GI Stromal Tumor Bearing a PDGFRA Deletion DIM842-844. J Clin Oncol..

[5] Liegl-Atzwanger B, Fletcher JA, Fletcher CDM (2010). Gastrointestinal stromal tumors. Virchows Archiv.

[6] Agaram NP, Wong GC, Guo T, Maki RG, Singer S, Dematteo RP, Besmer P, Antonescu CR (2008). Novel V600E BRAF mutations in imatinib-naive and imatinib-resistant gastrointestinal stromal tumors. Genes Chromosom Cancer.

[7] Boikos SA, Pappo AS, Killian JK, LaQuaglia MP, Weldon CB, George S, Trent JC, von Mehren M, Wright JA, Schiffman JD (2016). Molecular subtypes of KIT/PDGFRA Wild-Type gastrointestinal stromal tumors: a report from the national institutes of health gastrointestinal stromal tumor clinic. JAMA Oncol..

[8] Pantaleo MA, Nannini M, Corless CL, Heinrich MC (2015). Quadruple wild-type (WT) GIST: defining the subset of GIST that lacks abnormalities of KIT, PDGFRA, SDH, or RAS signaling pathways. Cancer Med.

[9] Sicklick JK, Lopez NE (2013). Optimizing surgical and imatinib therapy for the treatment of gastrointestinal stromal tumors. J Gastrointest Surg..

[10] Pantaleo MA, Nannini M, Corless CL, Heinrich MC (2015). Quadruple wild-type (WT) GIST: defining the subset of GIST that lacks abnormalities of KIT, PDGFRA, SDH, or RAS signaling pathways. Cancer Med..

[11] Nannini M, Astolfi A, Urbini M, Indio V, Santini D, Heinrich MC, Corless CL, Ceccarelli C, Saponara M, Mandrioli A (2014). Integrated genomic study of quadruple-WT GIST (KIT/PDGFRA/SDH/RAS pathway wild-type GIST). BMC Cancer.

[12] Frampton GM, Fichtenholtz A, Otto GA, Wang K, Downing SR, He J, Schnall-Levin M, White J, Sanford EM, An P (2013). Development and validation of a clinical cancer genomic profiling test based on massively parallel DNA sequencing. Nat Biotechnol.

[13] Liu X, Jian X, Boerwinkle E (2011). dbNSFP: a lightweight database of human nonsynonymous SNPs and their functional predictions. Hum Mutat.

[14] Liu X, Jian X, Boerwinkle E (2013). dbNSFP v2.0 a database of human non-synonymous SNVs and their functional predictions and annotations. Hum Mutat..

[15] Lek M, Karczewski KJ, Minikel EV, Samocha KE, Banks E, Fennell T, O’Donnell-Luria AH, Ware JS, Hill AJ, Cummings BB (2016). Analysis of protein-coding genetic variation in 60,706 humans. Nature.

[16] Grasso C, Butler T, Rhodes K, Quist M, Neff TL, Moore S, Tomlins SA, Reinig E, Beadling C, Andersen M (2015). Assessing copy number alterations in targeted, amplicon-based next-generation sequencing data. J Mol Diagn.

[17] Beadling C, Wald AI, Warrick A, Neff TL, Zhong S, Nikiforov YE, Corless CL, Nikiforova MN (2016). A multiplexed amplicon approach for detecting gene fusions by next-generation sequencing. J Mol Diagn.

[18] Rossi S, Sbaraglia M, Dell’Orto MC, Gasparotto D, Cacciatore M, Boscato E, Carraro V, Toffolatti L, Gallina G, Niero M (2016). Concomitant KIT/BRAF and PDGFRA/BRAF mutations are rare events in gastrointestinal stromal tumors. Oncotarget.

[19] Herz J, Strickland DK (2001). LRP: a multifunctional scavenger and signaling receptor. J Clin Investig.

[20] Prazeres H, Torres J, Rodrigues F, Pinto M, Pastoriza MC, Gomes D, Cameselle-Teijeiro J, Vidal A, Martins TC, Sobrinho-Simões M (2011). Chromosomal, epigenetic and microRNA-mediated inactivation of LRP1B, a modulator of the extracellular environment of thyroid cancer cells. Oncogene.

[21] Ni S, Hu J, Duan Y, Shi S, Li R, Wu H, Qu Y, Li Y (2013). Down expression of LRP1B promotes cell migration via RhoA/Cdc42 pathway and actin cytoskeleton remodeling in renal cell cancer. Cancer Sci.

[22] Liu C-X, Musco S, Lisitsina NM, Forgacs E, Minna JD, Lisitsyn NA (2000). LRP-DIT, a putative endocytic receptor gene, is frequently inactivated in non-small cell lung cancer cell lines. Cancer Res.

[23] Cowin PA, George J, Fereday S, Loehrer E, Van Loo P, Cullinane C, Etemadmoghadam D, Ftouni S, Galletta L, Anglesio MS (2012). LRP1B deletion in high-grade serous ovarian cancers is associated with acquired chemotherapy resistance to liposomal doxorubicin. Cancer Res.

[24] Colotta F, Allavena P, Sica A, Garlanda C, Mantovani A (2009). Cancer-related inflammation, the seventh hallmark of cancer: links to genetic instability. Carcinogenesis.

[25] Singh D, Chan JM, Zoppoli P, Niola F, Sullivan R, Castano A, Liu EM, Reichel J, Porrati P, Pellegatta S (2012). Transforming fusions of FGFR and TACC genes in human glioblastoma. Science.

[26] Gong Y, Zack TI, Morris LG, Lin K, Hukkelhoven E, Raheja R, Tan IL, Turcan S, Veeriah S, Meng S (2014). Pan-cancer genetic analysis identifies PARK2 as a master regulator of G1/S cyclins. Nat Genet.

[27] Veeriah S, Taylor BS, Meng S, Fang F, Yilmaz E, Vivanco I, Janakiraman M, Schultz N, Hanrahan AJ, Pao W (2010). Somatic mutations of the Parkinson’s disease-associated gene PARK2 in glioblastoma and other human malignancies. Nat Genet.

[28] Vasileiou G, Ekici AB, Uebe S, Zweier C, Hoyer J, Engels H, Behrens J, Reis A, Hadjihannas MV (2015). Chromatin-Remodeling-Factor ARID1B Represses Wnt/beta-Catenin Signaling. Am J Hum Genet.

[29] Wilson BG, Roberts CW (2011). SWI/SNF nucleosome remodellers and cancer. Nat Rev Cancer.

[30] Shiotani B, Zou L (2009). ATR signaling at a glance. J Cell Sci.

[31] Mao J, Kim BM, Rajurkar M, Shivdasani RA, McMahon AP (2010). Hedgehog signaling controls mesenchymal growth in the developing mammalian digestive tract. Development.

[32] Merchant JL, Saqui-Salces M (2014). Inhibition of Hedgehog signaling in the gastrointestinal tract: targeting the cancer microenvironment. Cancer Treat Rev.

[33] Krig SR, Miller JK, Frietze S, Beckett LA, Neve RM, Farnham PJ, Yaswen PI, Sweeney CA (2010). ZNF217, a candidate breast cancer oncogene amplified at 20q13, regulates expression of the ErbB3 receptor tyrosine kinase in breast cancer cells. Oncogene.

[34] Zhang ZC, Zheng LQ, Pan LJ, Guo JX, Yang GS (2015). ZNF217 is overexpressed and enhances cell migration and invasion in colorectal carcinoma. Asian Pac J Cancer Prev.

[35] Huynh KD, Fischle W, Verdin E, Bardwell VJ (2000). BCoR, a novel corepressor involved in BCL-6 repression. Genes Dev.

[36] Ghetu AF, Corcoran CM, Cerchietti L, Bardwell VJ, Melnick A, Privé GG (2008). Structure of a BCOR corepressor peptide in complex with the BCL6 BTB domain dimer. Mol Cell.

[37] Walker SR, Liu S, Xiang M, Nicolais M, Hatzi K, Giannopoulou E, Elemento O, Cerchietti L, Melnick A, Frank DA (2015). The transcriptional modulator BCL6 as a molecular target for breast cancer therapy. Oncogene.

[38] Cerchietti LC, Yang SN, Shaknovich R, Hatzi K, Polo JM, Chadburn A, Dowdy SF, Melnick A (2009). A peptomimetic inhibitor of BCL6 with potent antilymphoma effects in vitro and in vivo. Blood.

[39] Brenca M, Rossi S, Polano M, Gasparotto D, Zanatta L, Racanelli D, Valori L, Lamon S, Dei Tos AP, Maestro R (2015). Transcriptome sequencing identifies ETV6-NTRK3 as a gene fusion involved in GIST. J Pathol.

[40] Ornitz DM, Xu J, Colvin JS, McEwen DG, MacArthur CA, Coulier F, Gao G, Goldfarb M (1996). Receptor specificity of the fibroblast growth factor family. J Biol Chem.

[41] Zhang X, Ibrahimi OA, Olsen SK, Umemori H, Mohammadi M, Ornitz DM (2006). Receptor specificity of the fibroblast growth factor family. The complete mammalian FGF family. J Biol Chem.

[42] Hong DS, Brose MS, Doebele RC, Shaw AT, Dowlati A, Bauer TM, Farago AF, Estrada-Bernal A, Le AT, Cox MC et al. Clinical safety and activity from a phase 1 study of LOXO-101, a Selective TRK/A/B/C inhibitor, in solid-tumor patients with NTRK gene fusions. Proceedings of the 2015 AACR-NCI-EORTC International Conference on Molecular Targets and Cancer Therapeutics; 2015, Abstr PR13; 2015 Nov 5-9; Boston, Massachusetts.

[43] Gasparotto D, Rossi S, Polano M, Tamborini E, Lorenzetto E, Sbaraglia M, Mondello A, Massani M, Lamon S, Bracci R et al. Quadruple-negative GIST is a sentinel for unrecognized neurofibromatosis type 1 syndrome. Clin Cancer Res. 2016. PMID:27390349. 10.1158/1078-0432.CCR-16-0152 27390349

[44] Tang CM, Lee TE, Syed SA, Burgoyne AM, Leonard SY, Gao F, Chan JC, Shi E, Chmielecki J, Morosini D et al. Hedgehog pathway dysregulation contributes to the pathogenesis of human gastrointestinal stromal tumors via GLI-mediated activation of KIT expression. Oncotarget. 2016. PMID:27793025. 10.18632/oncotarget.12909 PMC5346634 27793025

[45] Wu Y-M, Su F, Kalyana-Sundaram S, Khazanov N, Ateeq B, Cao X, Lonigro RJ, Vats P, Wang R, Lin S-F (2013). Identification of targetable FGFR gene fusions in diverse cancers. Cancer discovery.

[46] Singh D, Chan JM, Zoppoli P, Niola F, Sullivan R, Castano A, Liu EM, Reichel J, Porrati P, Pellegatta S (2012). Transforming fusions of FGFR and TACC genes in human glioblastoma. Science (New York, NY).

[47] Cohen NA, Zeng S, Seifert AM, Kim TS, Sorenson EC, Greer JB, Beckman MJ, Santamaria-Barria JA, Crawley MH, Green BL (2015). Pharmacological inhibition of KIT Activates MET signaling in gastrointestinal stromal tumors. Cancer Res.

[48] Taipale M, Krykbaeva I, Whitesell L, Santagata S, Zhang J, Liu Q, Gray NS, Lindquist S (2013). Chaperones as thermodynamic sensors of drug-target interactions reveal kinase inhibitor specificities in living cells. Nat Biotechnol.

[49] Surveillance, Epidemiology, and End Results (SEER) Program (http://www.seer.cancer.gov) SEER*Stat Database: Incidence–SEER 18 Regs Research Data + Hurricane Katrina Impacted Louisiana Cases, Nov 2013 Sub (2000–2011) <Katrina/Rita Population Adjustment>- Linked To County Attributes–Total U.S., 1969–2012 Counties, National Cancer Institute, DCCPS, Surveillance Research Program, Surveillance Systems Branch, released April 2014 (updated 5/7/2014), based on the November 2013 submission.

