# Supplementary material for: FGFR1 and NTRK3 actionable alterations in “Wild-Type” gastrointestinal stromal tumors
Source: J Transl Med. 2016 Dec 14;14:339. doi: 10.1186/s12967-016-1075-6 (PMC5157084; doi:10.1186/s12967-016-1075-6)
Supplement: Supplementary file 1 — Additional file 1. Additional tables and figure. [file 12967_2016_1075_MOESM1_ESM.docx]

**Title**:  *FGFR1* and *NTRK3* Actionable Alterations in “Wild-Type” Gastrointestinal Stromal Tumors

**Authors:** Eileen Shi,^1^ Juliann Chmielecki,^2^ Chih-Min Tang,^3^ Kai Wang,^2^ Michael C. Heinrich,^4,5^ Guhyun Kang,^5,6^, Christopher L. Corless,^5^ Katherine E. Fero,^1,7^ James D. Murphy,^1,7^ Paul T. Fanta,^1,8^ Siraj M. Ali,^2^ Martina De Siena,^3^ Adam M. Burgoyne,^1,8^ Sujana Movva,^9^ Lisa Madlensky,^1,10^ David Hong,^11^ Gregory M. Heestand,^1,8^ Jonathan C. Trent,^12^ Razelle Kurzrock,^1,8^ Deborah Morosini,^2^ Jeffrey S. Ross,^2^ Olivier Harismendy,^1,13*^ and Jason K. Sicklick^1,3*^

^1^ School of Medicine, University of California San Diego, La Jolla, CA

^2^ Foundation Medicine, Inc., Cambridge, Massachusetts

^3^ Division of Surgical Oncology, Department of Surgery, Moores UCSD Cancer Center, University of California San Diego, La Jolla, CA

^4^ Portland VA Health Care System, Portland, OR

^5^ Oregon Health Sciences University, Knight Cancer Institute, Portland, OR

^6^ Department of Pathology, Sanggye Paik Hospital, Inje University, Seoul, Korea

^7^ UCSD Department of Radiation Medicine and Applied Sciences, Moores UCSD Cancer Center, University of California San Diego, La Jolla, CA

^8^ Division of Medical Oncology, Department of Medicine, Moores UCSD Cancer Center, University of California San Diego, La Jolla, CA

^9^ Department of Medical Oncology, Fox Chase Cancer Center, Philadelphia, PA

^10^ UCSD Department of Family and Preventive Medicine, Moores UCSD Cancer Center, University of California San Diego, La Jolla, CA

^11^ Department of Investigational Cancer Therapeutics, Division of Cancer Medicine, The University of Texas MD Anderson Cancer Center, Houston, TX

^12^ Sarcoma Medical Oncology Program, University of Miami Sylvester Cancer Center, Miami, FL

^13^ Division of Biomedical Informatics, Moores UCSD Cancer Center, University of California San Diego, La Jolla, CA

*** Co-Corresponding Authors:**

| Jason K. Sicklick, MD  Associate Professor of Surgery  Division of Surgical Oncology  Moores UCSD Cancer Center  University of California, San Diego  UC San Diego Health Sciences  3855 Health Sciences Drive, Room 2313  Mail Code 0987  La Jolla, CA 92093-0987  Tel: 858-822-3967  Fax: 858-228-5153  Email: [jsicklick@ucsd.edu](mailto:jsicklick@ucsd.edu) | Olivier Harismendy, PhD  Assistant Professor  Oncogenomics Laboratory  Moores UCSD Cancer Center  University of California, San Diego  UC San Diego Health Sciences  3855 Health Sciences Drive, Room 4335  Mail Code 0820  La Jolla, CA 92093-0820  Tel: [(858) 24**6-0248**](tel:858-246-0248)  Email: oharismendy@ucsd.edu |
| --- | --- |

**SUPPLEMENTAL TABLES**

**Supplemental Table S1: Annotation of Missense Variants of Unknown Significance.**

| **Number of Missense Variants of Unknown Significance (VUS)** | | |
| --- | --- | --- |
| Total | | 1240 |
| Identified in dbNSFP | | 1168 |
| Predicted Deleterious | SIFT ^1^ | 193 |
|  | PolyPhen ^2^ | 198 |
|  | MutationTaster ^3^ | 484 |
|  | MutationAssessor ^4^ | 280 |
|  | ≥2/4 (≥50%) Tools Predict Deleterious VUS | 325 |

^1^ Predicted Deleterious (D).

^2^ Predicted Deleterious (D).

^3^ Predicted Likely or Highly Deleterious.

^4^ Disease (D) or Disease Automatic (A).

**Supplemental Table S2: List of Variants also Present in ExAC Database at an Allele Frequency Greater than 1%.**

| **Gene** | **Mutation** | **Number of GIST Patients** | **Chr** | **Coord** | **RSID** | **Reference Allele** | **Alternate Allele** | **Allele Frequency** |
| --- | --- | --- | --- | --- | --- | --- | --- | --- |
| *NOTCH2* | P6fs*27 | 1 | 1 | 120612004 |  | CGG | C | AF=0.343 |
| *FANCD2* | N405S | 1 | 3 | 10088343 | rs73126218 | A | G | AF=0.412 |
| *MAP3K1* | S939C | 9 | 5 | 56177843 | rs45556841 | C | G | AF=0.015 |
| *MSH3* | A60_A62del | 2 | 5 | 79950724 | rs70991168 | G | GCCGCAGCGC | AF=0.039 |
| *ZNF217* | M410V | 1 | 20 | 52198138 | rs6068589 | T | C | AF=0.011 |

**Supplemental Table S3: Multiplexed Amplicon Approach for Detecting Gene Fusions by Next Generation Sequencing.**^1^

| **Target Kinase** | **Fusion Partners** |
| --- | --- |
| *AKT3* | *MAGI3* |
| *ALK* | *ATIC, C2orf44, CARS, CLTC, EML4, FN1, KIF5B, KLC1, MSN, NPM1, PPFIBP1, PTPN3, SEC31A, SQSTM1, STRN, TFG, TPM3, TPM4, TRAF1, VCL* |
| *BRAF* | *AGK, AGTRAP, AKAP9, CLCN6, FAM131B, FCHSD1, GNAI1, KCTD7, KIAA1549, MAD1L1, MKRN1, NUDCD3, PLIN3, RNF130, SLC45A3, SOX6, TRIM24, ZKSCAN5* |
| *EGFR* | *EGFR variant III, CAND1, PSPH, SEPT14, SLC12A9* |
| *ERBB4* | *EZR* |
| *ERG* | *TMPRSS2* |
| *FGFR1* | *BAG4, CPSF6, ERLIN2, PLAG1, TACC1, ZNF703* |
| *FGFR2* | *AFF3, AHCYL1, BICC1, CASP7, CCDC6, CIT, KIAA1967, OFD1, SLC45A3* |
| *FGFR3* | *BAIAP2L1, TACC3* |
| *MET* | *MIR548F1, TPR* |
| *NTRK1* | *BCAN, CD74, MIR548F1, MPRIP, NFASC, TFG, TPM3, TPR* |
| *NTRK2* | *NACC2, QKI* |
| *NTRK3* | *ETV6* |
| *NRG1* | *CD74, SLC3A2* |
| *PDGFRA* | *KDR, SCAF11* |
| *PDGFRB* | *NIN* |
| *RAF1* | *DAZL, ESRP1, MSS51, SRGAP3* |
| *RET* | *AFAP1, CCDC6, ERC1, HOOK3, KIAA1468, KIF5B, NCOA4, PARG, PCM1, PRKAR1A, TRIM27, TRIM33* |
| *ROS1* | *CCDC6, CD74, CEP85L, EZR, GOPC, KDELR2, LRIG3, SDC4, SLC34A2, TFG, TPM3* |

**Supplemental Table S4: Demographic and Clinicopathologic Data of 24 Wild-Type GIST** **Patients.**

| **Age (Years)** | **Sex** | **Primary Tumor Location** | **Tumor Stage** | **Biopsy Location^1^** | **Depth of NGS^2^ Coverage** |
| --- | --- | --- | --- | --- | --- |
| **Quadruple Wild-type GIST** | | | | | |
| 60 | Male | Small intestine | T3NxM1 | Small Intestine | 476 |
| 54 | Male | Stomach | T3N1M1 | Soft Tissue | 394 |
| 45 | Male | Small intestine | T3NxMx | Small Intestine | 476 |
| 47 | Female | Stomach | T4N0M1 | Liver | 651 |
| 44 | Male | Stomach | T3N0M1 | Stomach | 739 |
| 55 | Female | Stomach | T3N1M1 | Abdomen NOS | 433 |
| 55 | Male | Small intestine | T3N0M1 | Small Intestine | 463 |
| 60 | Female | Small intestine | T3N0Mx | Small Intestine | 393 |
| 26 | Female | Stomach | T3N0M1 | Stomach | 552 |
| 18 | Male | Stomach | T3N1M1 | Liver | 495 |
| 20 | Female | Stomach | T3N0M1 | Stomach | 493 |
| 44 | Male | Stomach | T3NxMx | Stomach | 626 |
| **Triple Wild-type GIST *** | | | | | |
| 38 | Female | Small intestine | T3N1M1 | Small Intestine | 829 |
| 41 | Male | Stomach | T4N1M1 | Diaphragm | 930 |
| 5 | Female | Small intestine | T2N0Mx | Abdomen NOS | 1018 |
| 42 | Male | Small intestine | T3N1M1 | Lymph Node | 757 |
| 57 | Female | Stomach | TxNxM1 | Colon | 629 |
| 58 | Female | Small intestine | T3N1M1 | Pancreas | 738 |
| 72 | Male | Small intestine | T4N1Mx | Small Intestine | 535 |
| 37 | Male | Stomach | T3N1Mx | Stomach | 606 |
| 47 | Female | Colon | T4N1M1 | Small Intestine | 1284 |
| 60 | Male | Colon | T3N0M1 | Stomach | 606 |
| 31 | Female | Stomach | T4N1M1 | Abdomen NOS | 848 |
| 50 | Female | Stomach | T2NxMx | Stomach | 768 |

^1^ NOS: not otherwise specified.

^2^ NGS: next-generation sequencing.

* patients with unknown SDHx mutation status due to use of a prior generation of CGP gene panel.

**Supplemental Table S5: Number and Type of Variants Observed in 186 GIST Patients (after VUS missense filtering).**

| **Type** | **Category (FM)** | **Type** | **Number** |
| --- | --- | --- | --- |
| Short Variants | known somatic | frameshift | 3 |
|  |  | inframe indel | 20 |
|  |  | Missense | 69 |
|  |  | nonsense | 9 |
|  |  | other | 11 |
|  | likely somatic | frameshift | 31 |
|  |  | inframe indel | 14 |
|  |  | Missense | 3 |
|  |  | nonsense | 23 |
|  |  | other | 15 |
|  |  | splicing | 14 |
|  | Variant of Unknown Significance | frameshift | 19 |
|  |  | inframe indel | 91 |
|  |  | Missense* | 915 |
|  |  | nonsense | 11 |
|  |  | other | 15 |
|  |  | splicing | 13 |
| Copy Number | known somatic | focal amplification | 23 |
|  |  | homozygous loss | 14 |
|  |  | amplification | 9 |
|  | observed | amplification | 34 |
|  |  | homozygous loss | 3 |
| Structural Variants | likely somatic | structural variant | 13 |
|  | observed | structural variant | 13 |

***VUS missense reported are predicted to be deleterious by 2/4 prediction tools (see Methods and Table S1)**

**Supplemental Table S6. List of mutations identified in *KIT/PDGFRA/SDH/RAS* pathways defining non-WT patients (in attached MS Excel file).**

**Supplemental Table S7. List of Mutations Identified in WT Patients (in attached MS Excel file).**

VUS are filtered according to Supplemental Tables S1 and S2.

**Supplemental Table S8: List of *FGFR1* alterations identified in the cohort.**

| **Patient Type** | **Gene Alteration** | **Class** |
| --- | --- | --- |
| WT - SDH unknown | FGFR1_HOOK3_fusion | Known deleterious |
| qWT | K656E | Known deleterious |
| qWT | FGFR1_TACC1_fusion | Known deleterious |
| non WT | amplification | Likely deleterious |
| non-WT | splice | VUS |
| non-WT * | S134D | VUS |
| non WT * | S134del | VUS |
| non-WT | V38M | VUS |

* Indicates mutations identified in the same tumor.

**Supplemental Table S9: Demographic and Clinicopathologic Data of 5 Wild-Type GIST** **Patients in the Secondary Study Population.**

| **Age (Years)** | **Gender** | **Primary Tumor Location** | **Tumor Stage** | **SDHB Immunostaining** | **Fusion Panel Result** |
| --- | --- | --- | --- | --- | --- |
| 54 | Male | Pelvic mass | Unknown | Unknown | *FGR1-TACC1* |
| 54 | Male | Colon | Unknown | Positive | *ETV6-NTRK3* |
| 49 | Male | Small intestine | T3NxMx | Positive | None detected |
| 51 | Female | Unknown | TxN1Mx | Positive | None detected |
| 53 | Male | Stomach | Unknown | Unknown | None detected |

**SUPPLEMENTAL FIGURE**

**Supplemental Figure 1: Deleterious Genomic Alterations, Genes and Tumor Sites in the Entire GIST Cohort (N=186).** Genomic alterations identified in 186 GIST, with each column representing an individual patient. This matrix is restricted to 9 genes defining canonical GIST drivers (*KIT, PDGFRA, NF-1, SDHA-D, BRAF,* *KRAS and HRAS*) and 120 genes mutated in WT GIST patients. VUS missense mutations are displayed only if they are predicted to affect gene function by 2 or more algorithms (see methods). Genes were prioritized on the basis of predicted damaging nature. The tumor site (blue scale in column header) is also indicated. See next page (8.5 x 14 inch page format).


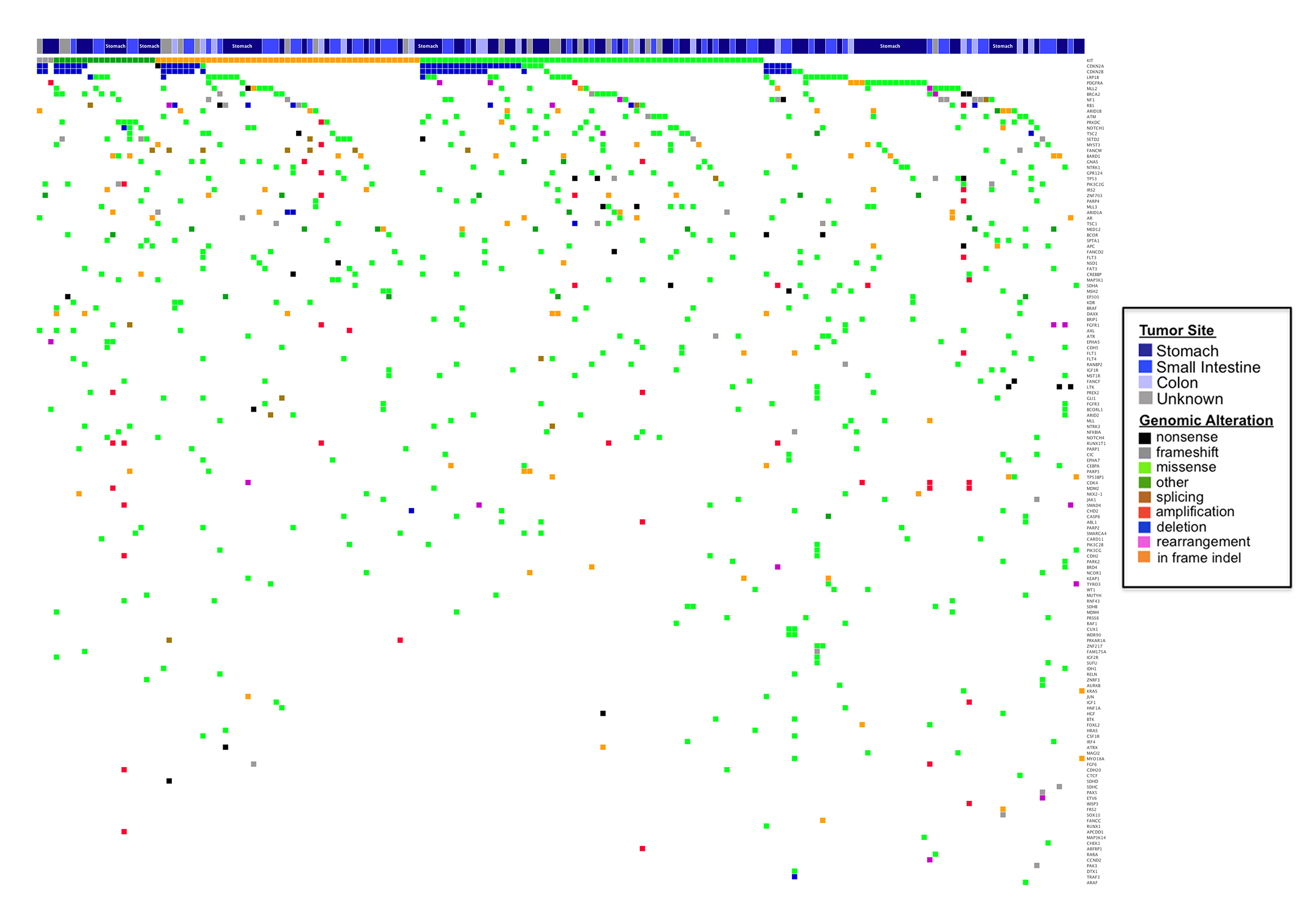


**SUPPLEMENTAL REFERENCES**

1. Beadling C, Wald AI, Warrick A, et al. A Multiplexed Amplicon Approach for Detecting Gene Fusions by Next-Generation Sequencing. J Mol Diagn 2016;18:165-75.
